# Clinical, Laboratory, and Radiographic Features Can Help Predict *Mycoplasma pneumoniae* Lower Respiratory Tract Infection in Children

**DOI:** 10.3390/microorganisms11051358

**Published:** 2023-05-22

**Authors:** Jasna Rodman Berlot, Špela Dolenc, Uroš Krivec, Darja Keše

**Affiliations:** 1 Department of Paediatric Pulmonology, University Children’s Hospital, University Medical Centre Ljubljana, 1000 Ljubljana, Sloveniauros.krivec@kclj.si (U.K.); 2 Faculty of Medicine, University of Ljubljana, 1000 Ljubljana, Slovenia; 3 Institute of Microbiology and Immunology, Faculty of Medicine, University of Ljubljana, 1000 Ljubljana, Slovenia

**Keywords:** *Mycoplasma pneumoniae*, respiratory tract infection, children

## Abstract

*Mycoplasma pneumoniae (Mp)* is a common cause of lower respiratory tract infection (LRTI) in children that is difficult to distinguish from LRTI of other etiologies. We aimed to determine if a combination of clinical, laboratory, and chest radiographic features can help identify patients at higher risk of *Mp* LRTI. We reviewed medical charts of children referred to our tertiary hospital with suspected acute mycoplasmal LRTI. Pharyngeal swabs obtained from patients were tested by *Mp* PCR. We compared epidemiological and clinical data of children with positive and negative *Mp* PCR results. In addition, a multivariable logistic regression analysis was performed to predict *Mp* LRTI based on the patient’s age, duration of symptoms, presence of extrapulmonary manifestations, laboratory findings, and chest radiographic findings. We included 65 children with *Mp* PCR-negative and 49 with *Mp* PCR-positive LRTI and no viral co-detection. Children with *Mp* LRTI were older (median age 5.8 vs. 2.2 years, *p* < 0.001), had a longer duration of symptoms on referral (median 7 vs. 4 days, *p* < 0.001), and lower median WBC (9.9 vs. 12.7 × 10^9^/L, *p* < 0.001). On chest radiograph, unilateral infiltrates were more frequently observed in the *Mp* PCR-positive group (57.5% vs. 24.1%, *p* = 0.001). Age, duration of symptoms, and chest radiographic findings had the highest predictive value for *Mp* LRTI in a multivariable logistic regression model. Our analysis suggests that a combination of clinical, laboratory, and chest radiographic features can be used to assess the likelihood of *Mp* LRTI and assist in decision-making for which children need further tests or macrolide antibiotic treatment.

## 1. Introduction

*Mycoplasma pneumoniae* (*Mp*) is an important cause of lower respiratory tract infections (LRTIs) in children and adults worldwide [1,2,3,4]. *Mp* LRTIs are usually mild and self-limiting, and patients are mostly managed in primary care [1]. Diagnosing *Mp* LRTI based on clinical and laboratory evaluation is often troublesome as it is difficult to distinguish it from LRTIs of other etiologies, especially viral ones [1,4,5]. Moreover, current diagnostic tests, including polymerase chain reaction (PCR) of upper respiratory tract specimens or serology, do not reliably differentiate between *Mp* infection and carriage [1,6]. Diagnostic testing is also often limited in the primary care setting. Therefore, physicians often prescribe macrolides without positive microbiology results, which results in an increase in macrolide-resistant *Mp* (MR*Mp*) and a parallel rise in macrolide resistance in other respiratory pathogens, which is observed worldwide [7,8,9].

With the challenges in diagnostic testing for *Mp* and the emergence of MR*Mp*, it is vital to determine clinical features to aid in diagnosing *Mp* LRTI in children. This study aimed to determine if a combination of clinical features, laboratory findings, and chest radiographic characteristics can help physicians identify patients at higher risk of *Mp* LRTI.

## 2. Materials and Methods

### 2.1. Study Design

We performed an observational retrospective study to determine whether a combination of clinical, laboratory, and radiographic features can help physicians identify patients at higher risk of *Mp* LRTI.

Characteristics of patients with LRTI that were PCR-positive for *Mp* in pharyngeal swabs during an *Mp* epidemic period in 2014 were compared with those of patients with LRTI that were PCR-negative for *Mp* in a non-epidemic period in 2012 [10]. Immunocompromised children and children with chronic pulmonary disease were excluded from the study because the underlying condition could affect the presentation and severity of the disease.

The data on age, gender, disease presentation, duration of symptoms on referral, laboratory biomarkers of inflammation, macrolide susceptibility, radiographic appearance, hospital admission, duration of hospital stay, need for oxygen therapy, and data related to complications and treatment were collected for all patients.

LRTI diagnosis was made based on physical examination revealing pathological lung auscultation and radiographic appearance consistent with a diagnosis of LRTI.

The National Medical Ethics Committee of the Republic of Slovenia approved the protocol for this study (No 0120-8/2018/4 and No 0120-244/2021/3).

### 2.2. Study Subjects

#### 2.2.1. *Mp pneumoniae* PCR-Positive LRTI Group

Children younger than 18 years, referred to University Children’s Hospital with suspected acute *Mp* LRTI from 1 January 2014 to 31 December 2014, were tested for *Mp* and respiratory viruses. All patients who were PCR-positive for *Mp* in pharyngeal swabs were identified from a laboratory database and included in the study. We excluded cases with viral co-detection.

#### 2.2.2. *Mp pneumoniae* PCR-Negative LRTI Group

Children younger than 18 years, referred to our tertiary hospital with suspected acute mycoplasmal LRTI from 1 January 2012 to 31 December 2012, were tested for *Mp*. All patients who were PCR-negative for *Mp* in pharyngeal swabs were identified from a laboratory database and included in the study. We excluded patients who did not have a nasopharyngeal swab for respiratory viruses performed.

### 2.3. Methods

Pharyngeal swabs to assess *Mp* infection were subjected to DNA isolation using automated MagNA Pure Compact System (Roche Diagnostics, Mannheim, Germany) and the MagNA Pure Compact Nucleic Acid Isolation Kit I (Roche Diagnostics, Mannheim, Germany). *Mp* real-time PCR was performed using the commercially available kit Chla/Myco pneumo r-gene (Argene BioMerieux diagnostics, Marcy l’Etoile, France) on a LightCycler 2.0 (Roche Diagnostics, Mannheim, Germany) platform according to the manufacturer’s instructions. Macrolide resistance was recognized by pyrosequencing two parts of domain V in the 23S rRNA gene [10].

Multiplex PCR using Respiratory Viruses 16-Well Assay V.17 (AusDiagnostics, Mascot, Australia) was performed on the nasopharyngeal swab specimens to assess viral co-detection, including respiratory syncytial virus, influenza virus, parainfluenza virus, human bocavirus, adenovirus, metapneumovirus, rhinovirus, enterovirus, and human coronavirus [11].

### 2.4. Analysis

Continuous variables were presented as mean (SD) or median (IQR), where appropriate. Categorical variables were described with counts and percentages. Continuous variables were compared using the independent samples *t*-test or the Mann–Whitney U-test, where appropriate. Categorical variables were compared using the Pearson Chi-Square test. A multivariable logistic regression analysis was performed to predict *Mp* LRTI based on epidemiological, clinical, laboratory, and radiographic features. The differences were considered statistically significant when the *p* value was <0.05. Statistical computing was conducted in IBM SPSS Statistics (Version 28.0).

## 3. Results

**Figure 1:**
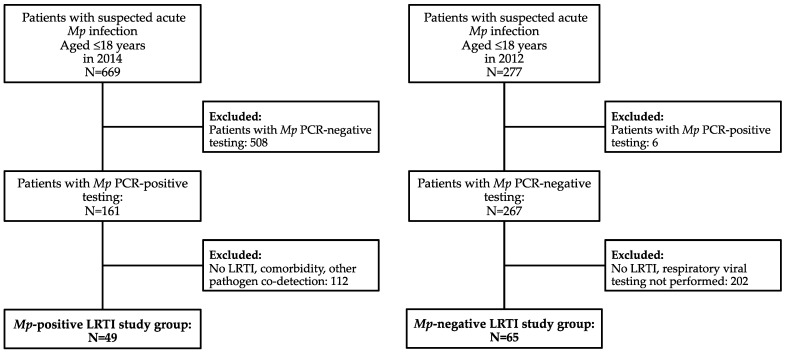
Study flowchart. Abbreviations: LRTI: lower respiratory tract infection; *Mp*: *Mycoplasma pneumoniae*.

In 2012 and 2014, 277 and 669 children were referred to University Children’s Hospital Ljubljana with a suspected *Mp* infection, respectively. Out of those, 2.2% (6/277) and 24.1% (161/669) tested *Mp* PCR-positive by pharyngeal swab in each study year, respectively. After applying the inclusion and exclusion study criteria, we evaluated and compared epidemiological and clinical data of 49 patients with *Mp* PCR-positive LRTI and no viral co-detection and 65 patients with *Mp* PCR-negative acute LRTI (Figure 1).

The characteristics of both groups of patients are detailed in Table 1. In the *Mp* PCR-negative LRTI group, 61.5% (40/65) of patients had a detected viral infection. Detected respiratory viruses included respiratory syncytial virus, influenza virus, parainfluenza virus, human bocavirus, metapneumovirus, rhinovirus, and coronavirus. Rhinovirus (40.0%, 16/40), respiratory syncytial virus (22.5%, 9/40), and human bocavirus (17.5%, 7/40) were the predominantly detected viruses. In the *Mp* PCR-positive group, all isolates (49/49) were macrolide susceptible.

Patients in the *Mp* PCR-positive group were older (median age 5.8 vs. 2.2 years, *p* < 0.001), with a longer duration of symptoms on referral (median 7 vs. 4 days, *p* < 0.001), and had higher median WBC levels than the *Mp* PCR-negative group (9.9 vs. 12.7 × 10^9^/L, *p* < 0.001) (Table 1). After *Mp* LRTI was PCR-confirmed, all patients received macrolide antibiotic treatment, while in the PCR-negative group, none received macrolide antibiotic treatment. X-ray was performed in 91.1% (41/45) of patients in the *Mp* PCR-positive and 84.6% (55/65) in the *Mp* PCR-negative group. Unilateral infiltrates were more frequently observed on chest radiograph in the *Mp* PCR-positive group (57.5% vs. 24.1%, *p* = 0.001), while bilateral infiltrates in the *Mp* PCR-negative group were more frequent (32.5% vs. 75.9%, *p* = 0.001) (Table 1). The most frequent extrapulmonary manifestations in the *Mp* PCR-positive LRTI group were the dermatological ones, observed in 12.2% (6/49) of cases, presenting as a non-specific maculopapular or urticarial rash. In comparison, gastrointestinal symptoms, such as vomiting and diarrhea were significantly more frequent in the *Mp* PCR-negative LRTI group (Table 1). Ophthalmological presentation, such as bilateral conjunctivitis, was observed only in the *Mp* PCR-negative group. Cardiovascular and neurological presentations were rare, observed in one patient with newly identified arrhythmia in the form of monomorphic ventricular extrasystoles in the rhythm of ventricular bigeminy in the *Mp* PCR-positive group, one patient with Guillain–Barre syndrome in the *Mp* PCR-positive group, and one patient with febrile convulsions in the *Mp* PCR-negative group.

The characteristics of hospitalized patients are summarized in Table 2. The two groups differed in the hospital admission rate, with patients in the *Mp* PCR-negative group requiring hospital admission more often (75.5% vs. 90.7%, *p* = 0.027). When comparing the characteristics of hospitalized patients, patients in the *Mp* PCR-positive group were older (median age 4.9 vs. 2.2 years, *p* < 0.001), with a shorter hospital stay (median 3 vs. 5 days, *p* < 0.001), and required oxygen treatment less often (54.1% vs. 86.4%, *p* < 0.001) (Table 2). No intensive care treatment was required in either group.

A multivariable logistic regression model showed that age, duration of symptoms on referral, and radiographic features had the highest predictive value for *Mp* PCR-positive LRTI (OR 1.28, 95% CI 1.08–1.52, *p* = 0.005, OR 1.15, 95% CI 1.01–1.31, *p* = 0.038, OR 3.15, 95% CI 1.02–9.72, *p* = 0.046, respectively). Older children, with a longer duration of symptoms and unilateral infiltrates on chest radiographs were more likely diagnosed with *Mp* PCR-positive LRTI (Table 3).

## 4. Discussion

*Mp* LRTI represents a significant healthcare burden as *Mp* is the most commonly detected bacteria in school-aged children hospitalized with community-acquired pneumonia (CAP) [3,12]. *Mp* LRTI can be challenging to distinguish from LRTI of other etiologies, especially from other atypical bacteria and viruses, resulting in frequent troublesome diagnosing of acute *Mp* infection [1,2,3,4,5]. This study evaluated epidemiological, clinical, laboratory, and radiographic features associated with *Mp* LRTI to help physicians identify patients at higher risk of *Mp* LRTI.

We compared the epidemiological and clinical characteristics of 49 patients with *Mp* PCR-positive and 65 patients with *Mp* PCR-negative LRTI. All patients had a high suspicion of mycoplasmal LRTI when tested for *Mp* by PCR. *Mp* PCR-positive patients were older, with a longer median duration of symptoms on referral and lower median WBC. This is consistent with the findings from a recent study which also showed that the proportion of children aged 3–7 years was significantly higher than that of other age groups (*p* < 0.05), suggesting that preschool children aged 3–7 years were a high-risk group for *Mp* LRTI [13]. Unilateral infiltrates were more frequently observed on chest radiographs in the *Mp* PCR-positive group. Moreover, dermatological manifestations were the most frequent extrapulmonary manifestation in the *Mp* PCR-positive group. At the same time, none experienced gastrointestinal symptoms, the most frequent extrapulmonary presentation in the *Mp* PCR-negative group. In addition, patients with *Mp* PCR-positive LRTI had a lower admission rate and need for oxygen treatment than the *Mp* PCR-negative group. Age, duration of symptoms, and radiographic features had the highest predictive value for *Mp* PCR-positive LRTI in a multivariable logistic regression model, with older children, with a longer duration of symptoms, and unilateral infiltrates on chest radiograph, more likely having *Mp* PCR-positive LRTI.

Expectedly, we observed a high infection rate with respiratory viruses in the *Mp* PCR-negative LRTI group, as viral LRTI can often mimic *Mp* LRTI [1,4,14,15,16]. This fact probably explains why the patients in our *Mp*-negative group were younger than those in the *Mp*-positive group. Several studies showed that viruses are an important cause of pneumonia in preschool children [1,4,14,15,16]. Moreover, a recent study suggested that viruses might play a vital role in making *Mp* pneumonia clinically apparent in this age group due to a high rate of viral co-detection observed in young children with *Mp* LRTI [14]. Nevertheless, almost half of the patients in our study were younger than five years in the *Mp*-positive group with no viral co-infection detected, supporting the idea that *Mp* is an important cause of LRTI in this age group as well.

Previous studies have investigated characteristics associated with *Mp* LRTI [3,17]. In comparison with these studies, where patients with *Mp* LRTI had a high rate of viral co-infection, we excluded those patients from the *Mp* PCR-positive LRTI group to better assess features only attributable to *Mp*. The U.S. Centers for Disease Control and Prevention (CDC) performed surveillance for radiologically confirmed CAP in hospitalized children and adults from 2010 to 2012 (EPIC Study), using PCR for the detection of *Mp* [3,12]. A viral or bacterial pathogen was detected in 81% of children, with multiple pathogens detected in 26% [3,12]. Whether such co-infection involving *Mp* with multiple other pathogens is related to the severity of illness in these patients is mostly unknown [1,18,19]. However, co-infection with other pathogens makes attributing specific features to *Mp* or other pathogens challenging [1]. Moreover, a previous study showed that mixed *Mp* infections with bacteria and viruses tend to be associated with more severe illnesses [18,19].

Similar to our study, children with *Mp* LRTI presented with significantly longer duration of symptoms on first referral compared with LRTI caused by other etiology in a recent study [17]. Moreover, patients with *Mp*-positive LRTI had statistically lower inflammatory biomarker levels than *Mp*-negative LRTI [17,20,21,22,23]. However, both our study groups had low median levels of WBC, which were in the normal range for the group’s median age, thus not making it an essential indicator of *Mp* infection.

*Mp* infections are usually mild or asymptomatic but they may sometimes develop into severe or life-threatening disease with severe dysfunction of the affected organs [1,2,3,4,5]. The complex pathogenic mechanisms of *Mp* result in a wide range of possible clinical outcomes [24,25,26]. The pathogenesis of related respiratory symptoms caused by *Mp* is mainly due to adhesion and direct damage, including nutrient predation, invasion and toxin, cytokine-induced inflammation damage, and immune evasion effect [26]. The pathogenesis of extrapulmonary manifestations on the other hand includes (1) direct damage mediated by invasion and inflammatory factors, (2) indirect damage caused by host immune response, and (3) vascular occlusion [26]. Although intrapulmonary and extrapulmonary manifestations are independent of each other, they share similarities such as direct invasion, and inflammatory- and immune-mediated damage [26]. Even though extrapulmonary manifestations can occur without respiratory symptoms, they usually present with a concomitant LRTI [1,24,25,27]. It is well-recognized that *Mp* can cause a broad array of extrapulmonary manifestations affecting almost every organ in the body [1,24,25]. Cutaneous disease is *Mp* infection’s most common extrapulmonary presentation [1,28,29]. Patients usually present with non-specific maculopapular and urticarial eruptions [28,29,30]. Even though skin disorders were the most frequent extrapulmonary manifestation in our *Mp* PCR-positive group, we found no statistical association with *Mp*. Nevertheless, this manifestation seems to increase the probability of an underlying *Mp* infection, as shown in previous studies [17]. In addition, our study shows that concomitant gastrointestinal symptoms, when having LRTI, significantly lower the risk of *Mp* LRTI.

Radiologic findings in *Mp* LRTI are usually variable, non-specific, and do not differentiate *Mp*-positive from *Mp*-negative CAP [1,2,3,4,5,31]. The most common findings on chest radiograph are peribronchial and perivascular interstitial infiltrates [1,2,3,4,5,31]. The EPIC study found multilobar bilateral infiltrates and pleural effusion more common among *Mp* CAP [3]. However, a high rate of viral co-detection could influence their results. We also found a higher incidence of bilateral infiltrates in patients in the *Mp* PCR-negative group, who frequently had a viral infection.

Clinical practice guidelines by the Pediatric Infectious Diseases Society and the Infectious Diseases Society of America recommend macrolide antibiotic treatment in patients with LRTI, with findings compatible with LRTI caused by atypical pathogens, such as *Mp* [32]. Therefore, physicians often prescribe macrolides without positive microbiology results [33]. The widespread macrolide usage has led to an increase in MR*Mp* isolates and a parallel rise in macrolide resistance in other respiratory pathogens, observed worldwide [7,8,9,34,35,36]. Macrolides are primarily bacteriostatic agents that bind to specific nucleotides in domains II and/or V of 23S rRNA in the 50S bacterial ribosomal subunit, blocking protein synthesis by causing premature dissociation of peptidyl-tRNA from the ribosome [37]. Several studies have demonstrated that macrolide resistance in *Mp* results from single point mutations in various positions in 23S rRNA [7,8,9,36]. Over 90% of *Mp* isolates are now resistant to macrolides in some regions of Japan and China [1,34]. In Europe, prevalence is substantially lower than in Asia and varies from country to country, with very low levels of 1% in our country [1,35,38]. Even though these results are very encouraging, surveillance for macrolide resistance is crucial because mycoplasmas have high mutation rates, and such mutational events can rapidly accumulate in a population where selection is taking place [34,35,36]. Moreover, macrolide resistance was documented even during the course of treatment by comparing strains isolated before and after initiation of macrolide therapy and confirming the presence of new mutations in the latter [39].

The global spread of MR*Mp* is becoming an important issue when treating *Mp* infections. The clinical relevance of MR*Mp* has been debated since the interplay of disease severity and resistant strain infection has yet to be clarified [9,36]. The presence of MR*Mp* does not change the clinical or radiographic presentation of pneumonia, so it is impossible to guess in advance who may be harboring resistant organisms [9,36]. Generally, antimicrobial resistance per se does not make a microorganism more virulent. However, it can make treatment more difficult and lead to complications when prescribed an ineffective antimicrobial agent [1,40]. Several studies have reported that individuals infected with MR*Mp* receiving macrolide treatment can experience a longer febrile period and more extended antibiotic therapy than those infected with macrolide-susceptible strains [1,40]. All *Mp* isolates in our *Mp* PCR-positive LRTI group were macrolide sensitive, therefore not a factor influencing our patients’ clinical presentation and disease severity.

Although *Mp* is endemically present in the population, increases in the number of infections have been reported to occur in localized outbreaks in closed settings and community-wide or even worldwide epidemics [1,34]. Epidemics encompassing broad geographic ranges tend to occur every few years, mostly at 3- to 7-year intervals [1,34]. Since 2010, several European countries have experienced increased numbers of *Mp* infections, Slovenia included [1,10,34]. It is unclear why epidemics occur in cyclic patterns, but one theory links this phenomenon to an alternation of *Mp* genotypes [1]. *Mp* isolates can be classified into two major genetic groups, designated subtype 1 and subtype 2, based on sequence differences in repetitive elements RepMP2/3 and RepMP4 in the P1 protein gene [1,41]. It has been speculated that the cyclical *Mp* epidemics that tend to occur every few years can be related to a shift from one P1 subtype to the other since the two major subtypes are immunologically distinct and exposure to one subtype may induce transient herd immunity that suppresses infection with that subtype while allowing the other one to reemerge [1]. Indeed, the alternating predominance of subtype 1 or subtype 2 strains in a population has been documented in longitudinal studies [10]. However, this is not always the case, as recent publications have shown co-circulation of the two P1 types and multiple variants during the same epidemic period, supporting the notion that epidemics are polyclonal in nature [10]. Physicians should be aware of potential ongoing *Mp* epidemics and outbreaks which increase the probability of acute *Mp* infection in patients with findings compatible with LRTI caused by atypical pathogens. In our study, the percentage of positive results increased from 2.2% to 24.1.%, from a non-epidemic to an epidemic period, respectively.

Current diagnostic tests for diagnosing acute *Mp* infection, including PCR of upper respiratory tract specimens or serology, do not reliably differentiate between *Mp* infection and carriage [1,6]. The most sensitive approach for early diagnosis of *Mp* infection would currently be a combination of IgM serology and real-time PCR in symptomatic patients [1]. This approach may be less accurate in adults with age-related impairment in immunity, resulting in low antibody responses after *Mp* infection [42]. The same situation may occur with very young infants who may be unable to mount a vigorous humoral immune response to *Mp* due to a reduced innate immune response in comparison to older children [42]. Moreover, the opportunity of performing two tests is usually very limited, especially in the primary care setting, where most *Mp* infections are being treated. As shown in our study, a combination of clinical features can help physicians identify patients at higher risk of *Mp* LRTI and limit testing to this patients’ group. The results of our study can provide a good foundation for a future prediction model for *Mp* LRTI.

The retrospective design of our study limited data collection to the most commonly used clinical variables, most of which were recorded in all patients. A prospective design would allow for assessing other characteristics associated with *Mp* LRTI. All our patients were recruited from a University hospital, which may have led to a disproportionate number of cases with more severe *Mp* LRTI, which can explain the high hospital admission rate and need for oxygen treatment observed in our study groups.

## 5. Conclusions

Serologic and molecular test results for *Mp* are rarely available in a clinically relevant time frame to influence outpatient antibiotic prescribing, leading to a potentially higher prevalence of MR*Mp*. Surveillance and rational macrolide prescribing is needed in the future in order to avoid macrolide resistance emergence. Our data support that although non-specific for *Mp* LRTI, some clinical, laboratory, and radiographic features may help physicians identify patients at higher risk for *Mp* LRTI and assist in decision-making for which children need further tests or macrolide antibiotic treatment. This warrants further investigations in a larger cohort of children with *Mp* infection.

## Figures and Tables

**Table 1:** Characteristics and comparison of *Mycoplasma pneumoniae* PCR-positive and *Mycoplasma pneumoniae* PCR-negative patients with lower respiratory tract infection. Data are presented as median (IQR) or as percentage (proportion of subjects). Significant differences (*p* < 0.05) are highlighted in bold.

	*Mp* PCR-Positive LRTI	*Mp* PCR-Negative LRTI	Test Statistic	*p* Value
Subjects N	49	65		
Boys/girls (%)	59.2/40.8	66.2/33.8	χ^2^ = 0.583	0.445
Age (years)	5.8 (IQR 3.4–8.6)	2.2 (IQR 1.3–4.5)	U = 729.00	**<0.001**
<5 years (%) 5–18 years (%)	44.9 (22/49)55.1 (27/49)	76.9 (50/65)23.1 (15/65)	χ^2^ = 4.89	**0.027**
Duration of symptoms (days)	7 (IQR 5–10)	4 (IQR 2–7)	U = 866.00	**<0.001**
CRP (mg/L)	13 (IQR 7–28)	17 (IQR 7–48)	U = 1423.50	0.539
WBC (×10^9^/L)	9.9 (IQR 7.0–11.6)	12.7 (IQR 9.3–15.6)	U = 935.00	**<0.001**
**Radiographic findings**				
Effusion (%)	22.0 (9/41)	21.8 (12/55)	χ^2^ = 0.00	0.988
Unilateral/Bilateral infiltrates (%)	57.5/32.5	24.1/75.9	χ^2^ = 10.87	**0.001**
**Extrapulmonary manifestations**				
All (%)	16.3 (8/49)	21.5 (14/65)	χ^2^ = 4.87	0.485
Dermatological (%)	12.2 (6/49)	4.6 (3/65)	χ^2^ = 2.24	0.135
Gastrointestinal (%)	0 (0/49)	10.8 (7/65)	χ^2^ = 5.62	**0.018**
Cardiovascular (%)	2.0 (1/49)	0 (0/65)	χ^2^ = 1.34	0.247
Neurological (%)	2.0 (1/49)	1.5 (1/65)	χ^2^ = 0.04	0.840
Ophtalmological (%)	0 (0/49)	3.1 (2/65)	χ^2^ = 1.54	0.215
Hospitalization (%)	75.5 (37/49)	90.7 (59/65)	χ^2^ = 4.89	**0.027**

Abbreviations: CRP: C-reactive protein; IQR: interquartile range; LRTI: lower respiratory tract infection; *Mp*: *Mycoplasma pneumoniae*; WBC: white blood cell count. Continuous variables were compared using Mann–Whitney U-test (Test statistic U), whereas categorical variables were compared by using Pearson Chi-Square test (Test statistic χ^2^).

**Table 2:** Characteristics of hospitalized patients with lower respiratory tract infection with either *Mycoplasma pneumoniae*-positive or *Mycoplasma pneumoniae*-negative PCR result. Data are presented as median (IQR) or as percentage (proportion of subjects). Significant differences (*p* < 0.05) are highlighted in bold.

	*Mp* PCR-Positive LRTI	*Mp* PCR-Negative LRTI	Test Statistic	*p* Value
Subjects N	37	59		
Age (years)	4.9 (IQR 3.2–8.1)	2.2 (IQR 1.3–4.6)	U = 542.00	**<0.001**
<5 years (%) 5–18 years (%)	51.4 (19/37) 48.6 (18/37)	76.3 (45/59) 23.7 (14/59)	χ^2^ = 6.36	**0.012**
Hospital stay (days)	3 (IQR 2–5)	5 (IQR 4–8)	U = 532.50	**<0.001**
Oxygen therapy (%)	54.1 (20/37)	86.4 (51/59)	χ^2^ = 12.38	**<0.001**

Abbreviations: IQR: interquartile range; LRTI: lower respiratory tract infection; *Mp*: *Mycoplasma pneumoniae.* Continuous variables were compared using Mann–Whitney U-test (Test statistic U), whereas categorical variables were compared by using Pearson Chi-Square test (Test statistic χ^2^).

**Table 3:** Multivariable logistic regression analysis for *Mycoplasma pneumoniae* PCR-positive lower respiratory tract infection in children. Significant differences (*p* < 0.05) are highlighted in bold.

	Odds Ratio (OR)	95% CI for OR	*p* Value
Age	1.28	1.08–1.52	**0.005**
Duration of symptoms	1.15	1.01–1.31	**0.038**
CRP	0.97	0.95–0.99	**0.012**
X-ray infiltrates *	3.15	1.02–9.72	**0.046**
Extrapulmonary manifestations	0.88	0.21–3.69	0.856
Hospitalization	0.29	0.04–2.21	0.233

* Unilateral vs. bilateral. Abbreviations: CRP: C-reactive protein.

## Data Availability

The data presented in this study are available on request from the corresponding author. The data are not publicly available due to privacy.
